# Mapping parental DMRs predictive of local and distal methylome remodeling in epigenetic F1 hybrids

**DOI:** 10.26508/lsa.202402599

**Published:** 2024-01-30

**Authors:** Ioanna Kakoulidou, Robert S Piecyk, Rhonda C Meyer, Markus Kuhlmann, Caroline Gutjahr, Thomas Altmann, Frank Johannes

**Affiliations:** 1 Plant Epigenomics, TUM School of Life Sciences Weihenstephan, Technical University of Munich , Munich, Germany; 2 Institute of Advanced Studies, Technical University of Munich , Munich, Germany; 3 Plant Genetics, TUM School of Life Sciences, Technical University of Munich, Freising, Germany; 4 Leibniz Institute of Plant Genetics and Crop Plant Research (IPK), Gatersleben, Germany

## Abstract

F1 hybrids derived from a cross between two inbred parental lines often display widespread changes in DNA methylation and gene expression patterns relative to their parents. An emerging challenge is to understand how parental epigenomic differences contribute to these events. Here, we generated a large mapping panel of F1 epigenetic hybrids, whose parents are isogenic but variable in their DNA methylation patterns. Using a combination of multi-omic profiling and epigenetic mapping strategies we show that differentially methylated regions in parental pericentromeres act as major reorganizers of hybrid methylomes and transcriptomes, even in the absence of genetic variation. These parental differentially methylated regions are associated with hybrid methylation remodeling events at thousands of target regions throughout the genome, both locally (in cis) and distally (in trans). Many of these distally-induced methylation changes lead to nonadditive expression of nearby genes and associate with phenotypic heterosis. Our study highlights the pleiotropic potential of parental pericentromeres in the functional remodeling of hybrid genomes and phenotypes.

## Abstract (precis)

We show that parental DNA methylation divergence in pericentromeric regions predicts non-additivity in the methylomes, transcriptomes, and phenotypes of F1 hybrids, independently of DNA sequence divergence.

## Introduction

Heterosis is a phenomenon in genetics in which the offspring of two inbred parents display superior trait performance (reviewed in Birchler et al [2010]). Although this phenomenon is extensively exploited commercially, its molecular mechanisms remain poorly understood (Chen, 2010). Genome-wide surveys of hybrids show that heterosis is accompanied by substantial nonadditive functional changes at the level of gene expression and epigenetic modifications, including DNA cytosine methylation (He et al, 2010; Groszmann et al, 2011; Chodavarapu et al, 2012; Greaves et al, 2012; Shen et al, 2012; Shen et al, 2017; Sinha et al, 2020). These functional changes appear to be because of dosage compensation in response to novel (epi)genetic combinations of the parental genomes being brought together in the progeny (Birchler et al, 2010). Interestingly, widespread functional remodeling can also be seen in hybrids whose parents are nearly isogenic (Greaves et al, 2012; Dapp et al, 2015; Rigal et al, 2016; Lauss et al, 2018), and experimental manipulation of parental DNA methylation pathways is sufficient to alter the heterotic potential of F1 progeny (Virdi et al, 2015; Yang et al, 2015; Kawanabe et al, 2016). Hence, in addition to genetic determinants, the epigenetic status of parental genomes appears to be an important factor in hybrid performance. Understanding the epigenotype–phenotype relation and the targeted use of epigenome diversity can contribute to breeding and increase crop production (Kakoulidou et al, 2021).

In plants, cytosine methylation occurs in sequence contexts CG, CHG, and CHH (where H = A, T, C). De novo methylation in all three contexts is primarily catalysed by the RNA-directed DNA methylation pathway, which involves 24 nucleotide (nt) small RNA (sRNA) that guide DOMAINS REARRANGED METHYLTRANSFERASE 2 (DRM2) to homologous target sequences (reviewed in Matzke and Mosher [2014]). The de novo activity of this pathway has been implicated in paramutation (also known as trans-chromosomal methylation) (Chandler, 2007; Greaves et al, 2012; Hövel et al, 2015), whereby hybrids display methylation gains in regions where the two parents are differentially methylated (Greaves et al, 2012). In this case, sRNAs are initially produced from the methylated parental allele, and subsequently targeted to the unmethylated allele for de novo methylation ([Chandler, 2007; Greaves et al, 2016]; reviewed in Greaves et al [2015]). Such remodeling events can lead to non-additive gene expression changes and are mechanistically well understood. However, most of the remodeling events in hybrids do not occur in parental differentially methylated regions (DMRs) (Greaves et al, 2012; Zhang et al, 2016; Lauss et al, 2018; Li et al, 2018; Ma et al, 2021; Kakoulidou & Johannes, 2023). Instead, they emerge in regions where the two parents are similarly methylated, and can involve both methylation gains and losses. These observations cannot be readily explained with classical paramutation models, but seem to depend on other mechanisms, including *distally* acting factors (Kakoulidou & Johannes, 2023). To date, there have been no systematic attempts to try to identify causal loci that facilitate these *distal* effects, partly because of limitations in experimental designs. Indeed, previous epigenetically informed studies of hybrids have examined relatively few crosses, which has precluded the use the of quantitative (epi)genetic approaches. As a result, it is currently unclear how genome-wide epigenetic remodeling in hybrids is linked to the emergence of heterosis at the phenotypic level.

**Figure 1.**
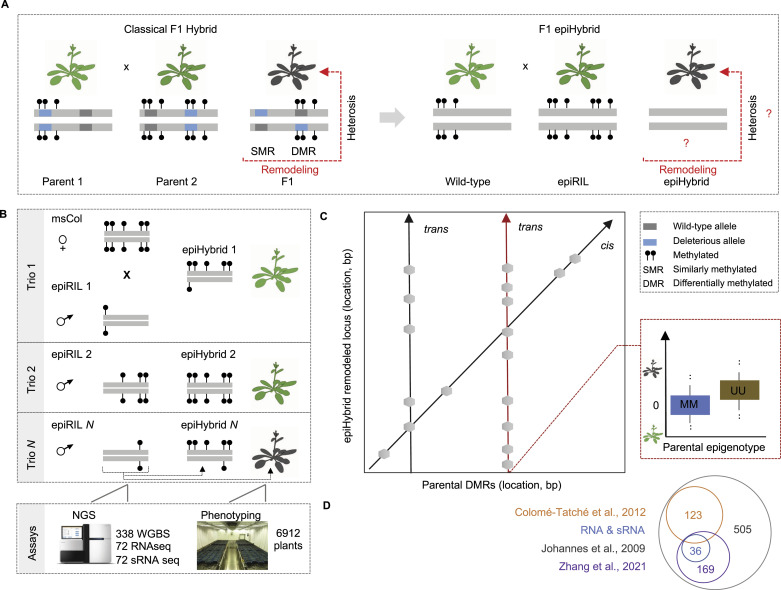
Schematic overview of the study. **(A)** Contrast between classical F1 hybrids (left) and F1 epiHybrids (right). In classical F1 hybrids, the two inbred parental lines differ both in fixed deleterious alleles and in their DNA methylation profiles. The confounding of these two sources of variation makes it difficult to delineate the causal mechanisms that lead to phenotypic heterosis and functional remodeling in their hybrid progeny. In F1 epiHybrids, the two parental lines are virtually isogenic but divergent at the DNA methylation level. These experimental systems can therefore be used to quantify epigenetic contributions to phenotypic heterosis and functional remodeling in their hybrid progeny. **(B)** Overview of the construction of the epiHybrid population. All plants were phenotyped and plant material was collected for whole-genome bisulphite sequencing, RNA and sRNA sequencing. Each trio is the combination of one maternal msCol line, one specific epiRIL line and the corresponding epiHybrid. **(C)** The epiHybrid experimental system permits systematic insights into how parental differentially methylated regions (x-axis) predict methylome rermodelling in the hybrids both locally (*cis*) and distally (*trans*) (left: *cis-trans* plot). We hypothesize that highly pleiotropic parental differentially methylated regions (x-axis) contribute to phenotypic heterosis of leaf are (y-axis) (right: boxplots). **(D)** A Venn diagram summarizing how the paternal epiRILs used in this study relate to the epiRILs used in previous studies.

A key methodological challenge is to study the relationship between parental and hybrid epigenomes in isolation from genetic variation. This is necessary to be able to attribute any functional and phenotypic changes seen in the hybrids to the epigenetic state of the parents, rather than to DNA sequence polymorphisms ([Johannes et al, 2009]; reviewed in Kakoulidou et al [2021] and Lloyd and Lister [2022]). To address this challenge, we analysed a large experimental system of F1 epigenetic hybrids (epiHybrids), whose parents are essentially isogenic but highly variable in their DNA methylation patterns (Fig 1A). Using a combination of multi-omic profiling and epigenetic mapping strategies (Fig 1), we show that parental DMRs are sufficient to facilitate the reorganization of hybrid methylomes and transcriptomes not only *locally*, but also *distally*. We find that pericentromeres of parental chromosomes harbour highly pleiotropic DMRs that induce targeted methylation and transcriptional changes at thousands of loci throughout the genome, most probably by way of distally acting sRNA. Importantly, these same DMRs are also predictors of phenotypic heterosis in this experimental system, and may serve as possible epigenetic breeding or editing targets in future applications.

## Results

### Construction of a large epiHybrid panel

**Figure S1.**
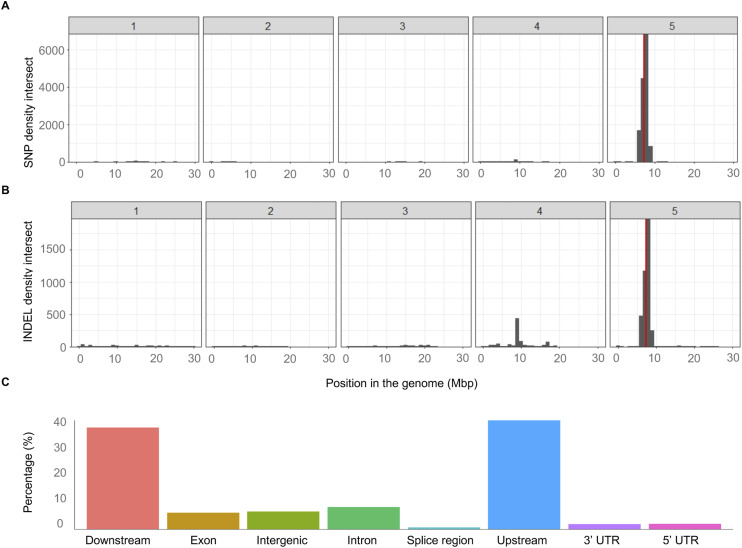
SNP calling in the msCol maternal line. **(A)** SNPs were called between each msCol line and the available Columbia ecotype genome to identify polymorphisms. The histograms show the SNP density for the intersection between msCol 12 and msCol 16 across the genome. **(B)** Histogram of INDEL density for the same intersection. Red lines indicate the locus of MS1 gene. **(C)** Bar plots shows the number of variants by effect region as the mean percentages for the two siblings (msCol 12 and msCol 16). Counts were extracted from the summary output of snpEff (Cingolani et al, 2012).

**Figure S2.**
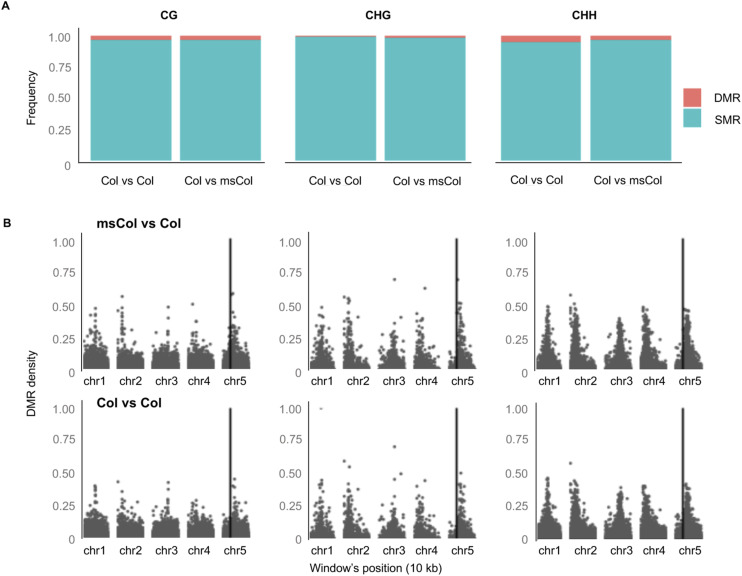
DMR calling in the msCol maternal line. **(A)** Differentially methylated regions were called between the msCol line and a publicly available Col-0 line (comparison indicated as Col versus msCol) and between the same Col-0 line and another Col line as a control (named as Col versus Col). **(B)** Histogram of differentially methylated region density across the genome for the two datasets. Black vertical line indicates the locus of MS1 gene.

We generated a large panel of 500 different *Arabidopsis thaliana* F1 epiHybrid families by crossing a male sterile maternal plant of the Columbia accession (msCol) to 500 different paternal *ddm1-2*-derived epiRILs (Johannes et al, 2009) (see the Materials and Methods section) (Fig 1B). msCol was chosen because it does not produce viable pollen due to a mutation in *MALE STERILITY 1* (*MS1*), and thus reduces crossing errors arising from hand pollination or unwanted self-fertilisation. The *ms1* allele was originally derived in a Landsberg (Ler) background and subsequently introgressed into Columbia by six generations of repeated backcrossing (Melchinger et al, 2007). Whole-genome re-sequencing (see the Materials and Methods section) confirmed a homozygous ∼2.7 Mb introgressed Ler segment around the MS1 locus on the arm of chromosome 5 (7.36–7.37 Mb) and only a small number of homozygous, mainly noncoding, SNPs and small INDELs outside of that region (Fig S1). Similarly, we found that the msCol methylome strongly resembles that of a Col WT plant (see the Materials and Methods section) (Fig S2). Hence, the introgression of the *ms1* allele did not have any detectable effects in distal locations on DNA methylation patterns outside of the introgressed region.

**Figure S3.**
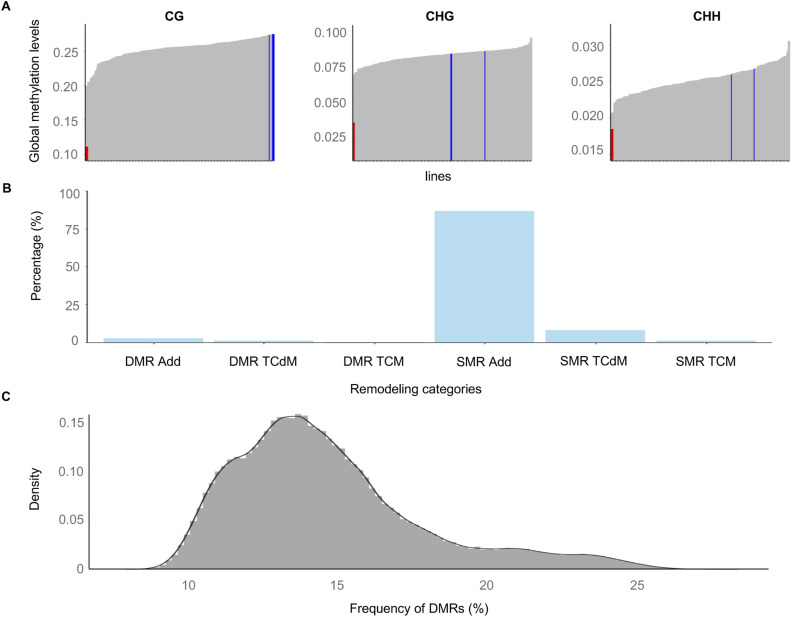
DNA methylome profiling of the epiHybrids. **(A)** Global methylation levels of each line. Grey bars represent global methylation level of each paternal epiRIL line; red bars *ddm1-2* and blue bars the msCol maternal lines. **(B)** Bar plot shows the proportions of regions displaying each remodeling category. NAD regions are the trans-chromosomal methylation and trans-chromosomal de-methylated events both for similarly methylated regions and differentially methylated regions (DMRs). **(C)** Density plot shows the DMR frequency distribution for all remodeling regions in bootstrap samples across 169 epiHybrid lines. We performed 1,000 times a permutation test to randomly select remodeling regions and then, calculate the frequency of NAD-DMRs within selected NADs (see the Materials and Methods section).

By contrast, the paternal *ddm1-2*-derived epiRILs (henceforth epiRILs) differ substantially in DNA methylation patterns from Col WT (Fig S3A), although their DNA sequence background is nearly identical (Johannes et al, 2009). They segregate thousands of hypomethylated regions across the genome, which were originally induced by a transient mutation in the ATPase chromatin remodeler *DECREASE IN DNA METHYLATION 1* (*DDM1*) (Johannes et al, 2009; Colome-Tatche et al, 2012; Zhang et al, 2021). Previous work showed that these *ddm1-2*-induced methylation losses contribute to the heritability of a broad range of complex traits, including plant height, flowering time, root length, and biotic stress responses (Roux et al, 2011; Zhang et al, 2012; Latzel et al, 2013; Cortijo et al, 2014; Kooke et al, 2015, 2019; Lauss et al, 2018; Furci et al, 2019).

Although the hypomethylation of the epiRIL genomes has also been linked to the de-repression and increased mobilisation rates of some TE families (CACTA, *ATCOPIA93*, *ATENSPM3 VANDAL21*) (Johannes et al, 2009; Cortijo et al, 2014; Quadrana et al, 2019), short-read re-sequencing indicated that such mobilisation events are rare and mainly private to each epiRIL (Johannes et al, 2009; Marí-Ordóñez et al, 2013; Cortijo et al, 2014; Quadrana et al, 2019). In addition, there is no indication that the epiRILs segregate many other types of genetic variants, such as SNPs or INDELs, that may have originated from the initial *ddm1-2* founder (Lauss et al, 2018). The exception is a well-described 2 Mbp inversion on chromosome 2 (Zhang et al, 2023), and possibly a few medium-sized duplications that have recently been detected using long-read sequencing of different *ddm1* siblings (Yi & Richards, 2008; Zhang et al, 2023).

Hence, in our experimental design, the parental lines of each epiHybrid family are nearly isogenic at the DNA sequence level (Col-wt background), but differ substantially in their DNA methylation profiles. By using the msCol line as the same maternal parent, our design further ensures that any molecular or phenotypic variation among epiHybrid-families can only originate from the methylome contributions of the paternal chromosome, as the maternal copy is shared by all F1 families. This allowed us to assess systematically if and how particular regions of the paternal methylomes contribute to heterosis among the epiHybrids, both at the molecular and phenotypic levels.

### Patterns of local methylome remodeling in epiHybrids

**Figure 2.**
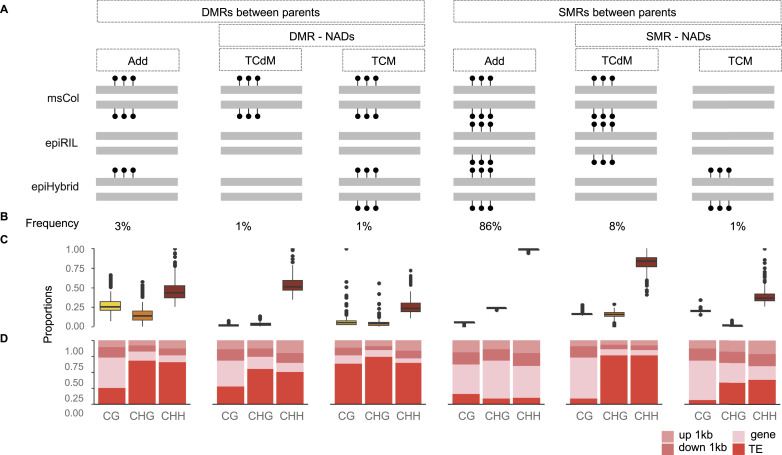
Categorizing DNA methylation remodeling in the epiHybrids. **(A)** Schematic model used to categorize DNA methylation remodeling regions. The genome was divided into 200-bp regions. Each region was defined as either a differentially methylated region or similarly methylated region, depending on the methylation status of the two parents. By comparing the region–level methylation states of the epiHybrids with those of the two parents, each region was further classified as additive (ADD), trans-chromosomal methylated, or trans-chromosomal de-methylated. Trans-chromosomal methylation and trans-chromosomal de-methylated regions were collectively defined as non-additive’ (NAD) regions. **(B)** Genome-wide frequency of each remodeling category. **(C)** Remodeling categories partitioned by cytosine context. **(D)** Annotation enrichment within each remodeling category.

**Figure S4.**
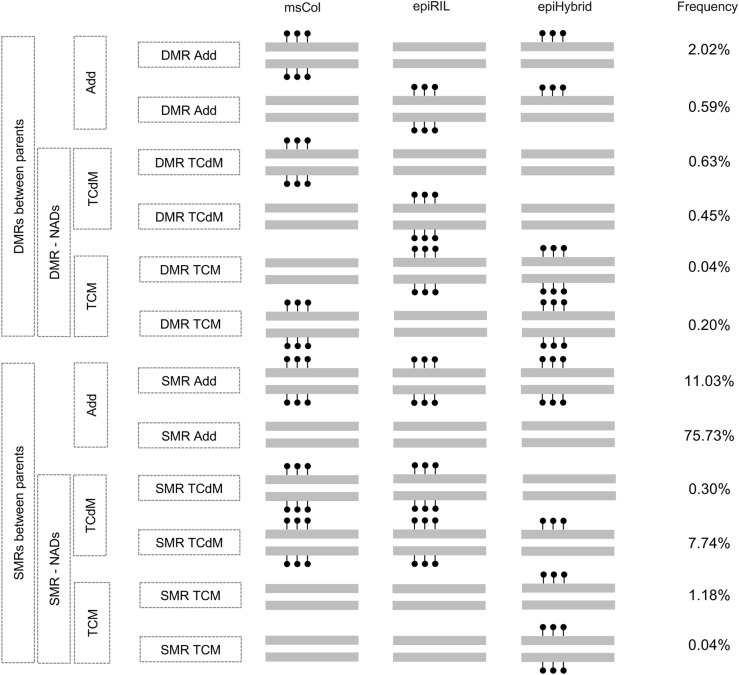
Extension of the schematic model used to categorize DNA methylation remodeling regions (from Fig 2A). The genome was divided into 200-bp regions. Each region was defined as either differentially methylated region or similarly methylated region, depending on the methylation status of the two parents. By comparing the region-level methylation states of the epiHybrids with those of the two parents, each region was further classified as additive (ADD), trans-chromosomal methylated, or trans-chromosomal de-methylated. Trans-chromosomal methylation and trans-chromosomal de-methylated regions were collectively defined as‚ non-additive’ (NAD) regions.

We performed whole-genome bisulphite sequencing (WGBS) for 169 epiHybrids (pooled siblings) and their parents (382 samples in total). The paternal epiRIL methylomes from this experiment were recently published in Zhang et al (2021), but are integrated here (Zhang et al, 2021). For each sequence context separately (CG, CHG, and CHH), we partitioned the genome into 200-bp regions (step size 50 bp) and compared the methylation status of each epiHybrid with that of its two parents. Based on this comparison, we classified each region into one of five different categories using the terminology of Zhang et al (2016): Additivity, TcM-DMR, TcdM-DMR, TcM-SMR, and TcdM-SMR (Zhang et al, 2016) (see the Materials and Methods section) (Figs 2A and S4) (Tables S1, S2, S3, S4, S5, and S6). Consistent with previous reports (Greaves et al, 2012; Shen et al, 2012; Groszmann et al, 2014; Dapp et al, 2015; Rigal et al, 2016; Zhang et al, 2016; Lauss et al, 2018; Sinha et al, 2020; Ma et al, 2021), a substantial proportion of regions (11% on average) displayed nonadditive methylation (NAD) in the epiHybrids (Figs 2B, S3B, and S4); that is, their methylation status diverged from what would be expected had the methylation status of paternal and maternal alleles been stably inherited. Most of these NADs (∼87%) occurred in regions where both parents were similarly methylated regions (SMRs) (Table S5). These NADs were highly enriched for CHH sites within TEs (see the Materials and Methods section) (Fig 2C and D), which explains their preferential co-location in pericentromeric regions of chromosomes (see the Materials and Methods section) (Fig 4A). By contrast, only about 13% of all NADs occurred in regions where the parents were DMRs (Figs 2B and S3B) (Table S5). However, considering that only 4% of the parental genomes are DMRs, on average, the occurrence of NADs within these regions constitutes a substantial enrichment (see the Materials and Methods section) (Fig S3C) (bootstrap test, *P*-value < 0.0001). Indeed, 34% of all parental DMRs were remodeled in the epiHybrids compared with 10% of all SMRs.

Table S1 Summary of sequencing statistics (sequencing depth and genome coverage) from the whole-genome bisulphite sequencing Methylstar pipeline.

Table S2 Regions identified using the sliding window method in the jDMR toolkit for CG context.

Table S3 Regions identified using the sliding window method in the jDMR toolkit for CHG context.

Table S4 Regions identified using the sliding window method in the jDMR toolkit for CHH context.

Table S5 Description of scenarios for finding differentially methylated regions and similarly methylated regions among jDMR regions, including the calculation of occurrence statistics (mean and SD) across all lines and the overall frequency for each type of region.

Table S6 Sample output from the jDMR pipeline for an epiHybrid line, showing methylation levels in parents and hybrids, along with the scenario definition based on the details in Table S5.

**Figure 3.**
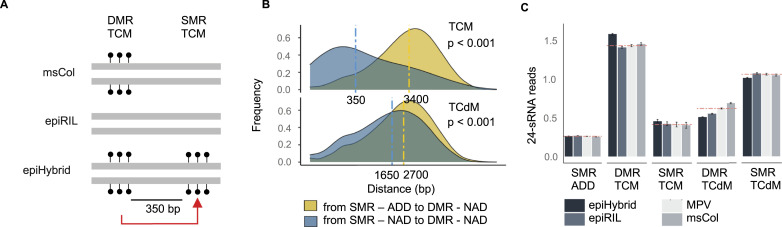
Integrated analysis of the remodeling scenarios. **(A)** A schematic showing how a differentially methylated region-trans-chromosomal methylation (DMR-TCM) event leads to a TCM event in a proximal similarly methylated region (SMR). **(B)** The plot indicates the frequency of distances for DMR-TCM and DMR-TCdM regions. Upper plot: yellow color indicates the distance of a given SMR-ADD region to a DMR-TCM region, whereas the blue color indicates the distance of a given SMR-TCM region to a DMR-TCM region. Bottom plot: yellow indicates the distance of a given SMR-ADD region to a DMR- TCdM region, whereas blue indicates the distance of a given SMR-TCdM region to a DMR-TCdM region. Vertical lines correspond to the median of each group. **(C)** Barplots showing normalized 24-sRNA read counts for each category for CHH context. Horizontal line indicates the mean value of the middle—parental value.

As described above, a well-characterised DMR-associated remodeling event is trans-chromosomal methylation (TCM), whereby the epiHybrids display local methylation gains (Greaves et al, 2012). This process involves 24 nt small RNAs that are initially produced from the methylated parental allele, but are subsequently targeted to the unmethylated allele for de novo methylation (Chandler, 2007; Greaves et al, 2012; Shivaprasad et al, 2012). To test for an sRNA involvement in the TCM classified regions, we performed small RNA sequencing for 36 epiHybrids and their parental lines (72 samples in total) (see the Materials and Methods section). Our analysis showed that TCM-DMRs are clearly accompanied by a local increase in 24 nt sRNA abundance in the epiHybrids, with levels either equalling or exceeding those of the homozygous methylated parent (Fig 3C). Similarly, we found that trans-chromosomal *de*-methylation (TCdM) at parental DMRs (TCdM-DMRs) correlates with a substantial reduction in 24 nt sRNA levels, although the mechanisms by which small RNAs are locally lost remains unclear (Zhang et al, 2016).

**Figure S5.**
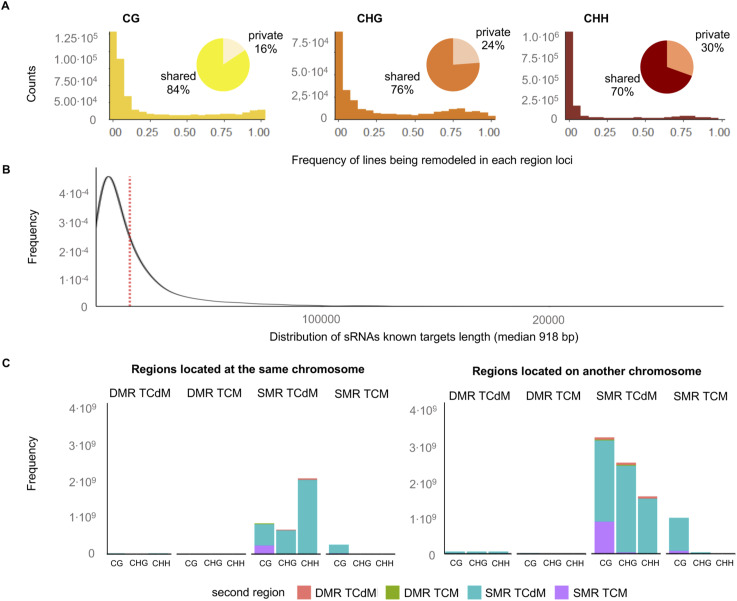
Population-level properties of DMRs in the epiHybrids. **(A)** The histogram indicates the frequency of each lines being a NAD (a remodeling case) in each 200-bp region. A frequency of 1 indicates that all 169 lines are NADs at one specific 200-bp region. A private region is when it is remodeled in only one epiHybrid line, and as shared if it is remodeled in at least two lines. **(B)** Distribution of sRNAs known targets length (median 918 bp). **(C)** Composition of significant NAD correlations. On the left, bar plots shows the frequency of pairs of regions where both correlating regions are located at the same chromosome. On the right, the bar plot shows the frequency of the pairs of regions where the correlating regions are located on different chromosomes. In the x-axis is indicated the identity of the first region of each pair, whereas filled with color indicates the identity of the second correlating region.

We explored if a similar sRNA association could be observed for TCM events occurring within SMRs (i.e., epiHybrid gain in regions where both parents are unmethylated). One hypothesis is that such TCM–SMRs are the result of sRNA-mediated spreading of DNA methylation from TCM events at proximal DMRs (Kakoulidou & Johannes, 2023) (Fig 3A). Our data support this hypothesis: We found that TCM–SMRs are only about 350 bp away from TCM-DMRs, on average, and display similar sRNA changes. Because the typical 24 nt sRNA cluster is about 918 bp in length (Fig S5B), it is likely that many TCM–SMR events are simply a by-product of sRNA changes at neighbouring TCM–DMRs. A similar trend could be observed for TCdM events within SMRs (i.e., epiHybrid loss in regions where both parents are methylated), but was much less pronounced, with the closest TCdM–DMR being relatively far way (1,650 bp, on average) (Fig 3B). This later observation suggests the involvement of other, possibly distally-acting, factors in mediating TCdM–SMR events.

### Genome-wide co-remodeling of distal regions is widespread among the epiHybrids

**Figure 4.**
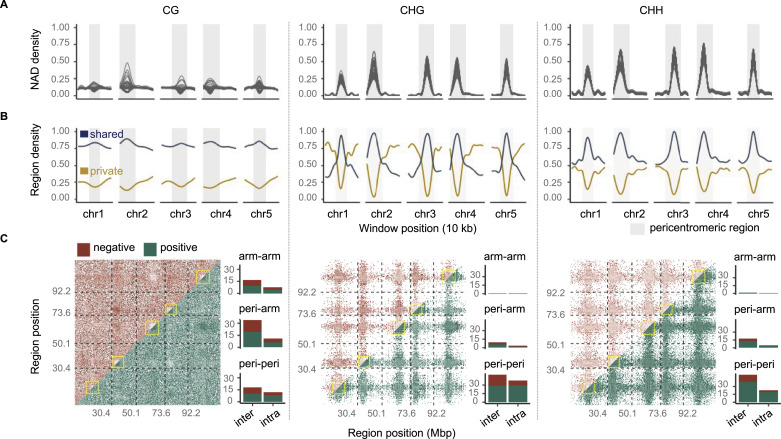
Patterns of methylome (co)-remodeling in the epiHybrids. **(A)** Genome-wide density of NAD regions in 10 kb sliding windows (step size 10 kb). Each line represents one epiHybrid family; grey areas indicate pericentromeric regions. **(A, B)** Genome-wide density of shared (blue) and private (yellow) NAD regions in the same sliding windows as in (A). **(C)** Frequency density of genome-wide negative (upper triangle) and positive (lower triangle) significant (*P*-value < 0.05) pairwise correlations between NAD regions. Only NADs that are shared by least 10 epiHybrid families are considered. Color intensity indicates levels of significance. The barplots give the frequency of significant intra- and inter-chromosomal correlations, belonging to the indicated categories. Category “arm-arm”: Both NAD regions are located in chromosome arms; category “arm-peri”: One NAD region is located in the chromosome arm, but the other in the pericentromeric region; category “peri-peri”: Both NAD regions are in the pericentromeric region.

**Figure S6.**
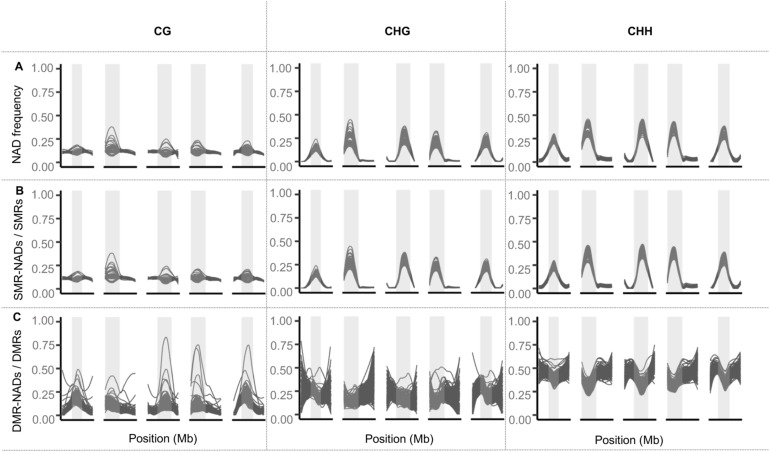
Genome-wide distribution of NADs in the epiHybrids. **(A)** Genome-wide density of NAD regions in 10-kb sliding windows (step size 10 kb). Each line represents one epiHybrid family. **(B)** Genome-wide density of similarly methylated region NADs among similarly methylated regions in 10 kb sliding windows (step size 10 kb). Each line represents one epiHybrid family. **(C)** Genome-wide density of differentially methylated region NADs among differentially methylated region regions in 10-kb sliding windows (step size 10 kb). Each line represents one epiHybrid family.

Unlike previous studies that focused on epigenomic data of single or few hybrid crosses, our multi-family experimental design allowed us to correlate NADs between any two regions across the genome. This gave us an opportunity to identify co-occurring TCdM and TCM events between distal regions. To facilitate such an analysis, we focused on NADs that were shared by at least 10 epiHybrid families (Fig S5A). The proportion of such shared NADs was substantial (21% for CG, 15% for CHG, and 23% for CHH), indicating that local remodeling events are a reproducible, rather than a random feature of the epiHybrid genomes. Shared NADs were highly enriched in pericentromeric regions of chromosomes, in contrast to NADs that were private to each epiRIL (Figs 4B and S6). Using these shared NADs, we quantified the extent of midparent methylation divergence (in %) for a given region in each epiHybrid, and used this measure as a quantitative molecular trait for pairwise correlation analysis (see the Materials and Methods section). Strikingly, this analysis uncovered a marked correlation structure across the genome, resembling a “checker-board pattern” (Fig 4C). Most notable were the strong positive correlations within and across pericentromeric regions of chromosomes, mainly in the context CHG and CHH. For context CG, these correlations were more evenly distributed across the genomes (Fig 4C).

**Figure S7.**
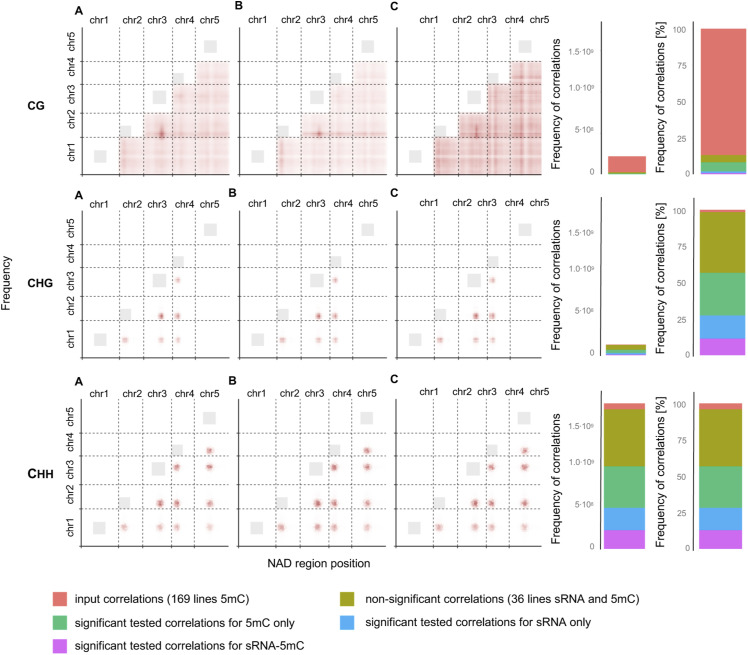
Correlation structure of the distally located regions. **(A, B, C)** The scatter plots are showing the frequency density of the significant correlations for selected inter-regions in sRNA and methylation (A), sRNA (B) and methylation (C); for each context separately. On the right, the stacked barplots are showing the frequency of correlations, which were tested for 5 mC, but not for sRNA because of the small number of samples (input correlation), correlations significant either for sRNA only, 5 mC only or sRNA and 5 mC, and correlations which were not significant for sRNA and 5 mC.

We decomposed the NAD correlation structure further, and discovered that most correlated regions harboured TCdM-SMR events that are affected by other remodeling events in distal regions, often located on another chromosome (see the Materials and Methods section) (Fig S5C). To relate these distal events to sRNA activity, we further correlated the mid-parental methylation divergence in 24 nt sRNA abundance (in %) of these same regions, and found a similar correlation structure at the sRNA level (see the Materials and Methods section) (Fig S7). This correlative evidence suggests that distally coordinated NAD events are at least partly the result of these regions being co-targeted by distally acting sRNA.

### Parental DMRs direct methylome remodeling locally and at distal regions

**Figure 5.**
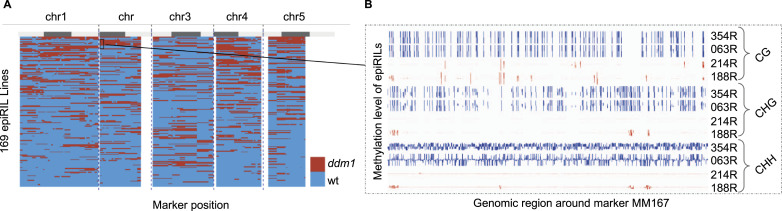
Epihaplotypes in the 169 paternal epiRILs. **(A)** A haplotype map of the 169 parental epiRIL. Each row represents one epiRIL. Haplotypes were inferred by using 144 segregating differentially methylated regions as molecular markers (Zhang et al, 2021). For each differentially methylated region, a given epiRIL can be either epihomozygous for the WT methylated state (MM, blue) or epihomozygous for the *ddm1*-like state (UU, red). Gray bars on top indicate the chromosome arms (light) and pericentromeric regions (dark). **(B)** An example genome browser view (IGV, Robinson et al, 2010) of four paternal epiRIL at the MM167 marker position. Blue color indicates epiRILs that are hypermethylated at these loci, and red color indicates hypomethylated epiRILs.

**Figure S8.**
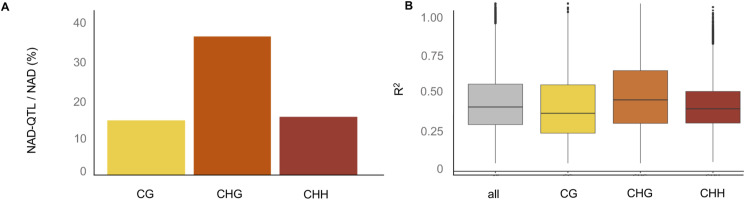
Properties of NAD-QTL targets and NAD-QTL effect sizes. **(A)** Barplot showing the average frequency of significant NAD-QTL targets among all NADs for each context. **(B)** Coefficient of determination (R2) for the linear regression model %MPDiv ∼ epigenotype.

The fact that DNA methylation and sRNA remodeling events are recurrent in so many independent epiHybrid families indicated to us that they could be traced back to methylome features that are shared among the paternal parents. As mentioned above, the paternal epiRILs segregate hypomethylated haplotypes across the genome, especially within pericentromeres of each chromosome (see the Materials and Methods section) (Fig 5A and B). Because of the experimental setup, these segregating regions are shared by about 25% of the epiRILs, the rest being WT methylated (Johannes et al, 2009; Colome-Tatche et al, 2012; Zhang et al, 2021). To explore if these regions can be used to predict remodeling in the epiHybrids, we employed an epigenetic quantitative trait locus (QTL^epi^) mapping strategy (Cortijo et al, 2014) (see the Materials and Methods section). In this approach, we used segregating parental DMRs as markers (predictors) and the degree of midparental methylation divergence (in %) at each NAD in the epiHybrids as a molecular quantitative trait. Akin to expression QTL mapping, we thus performed one genome-wide linkage scan for each shared NAD. This allowed us to assess if a given QTL^epi^ associates with a specific NAD locally or at distal region (Fig 1C). Our analysis revealed that 15%, 38%, 16% of all CG, CHG, and CHH NADs were associated with a QTL^epi^, respectively (Fig S8A). These QTL effects were substantial, explaining on average 38% of the mid-parent methylation divergence in each associated NAD region (Fig S8B) (see the Materials and Methods section).

**Figure 6.**
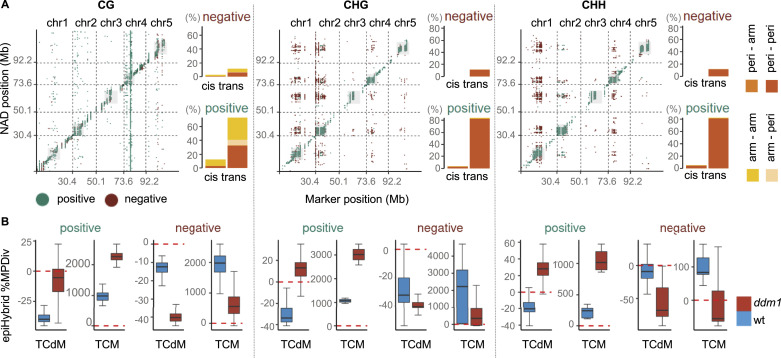
Parental differentially methylated regions direct methylome remodeling both locally (*cis*) and distally (*trans*). **(A)** Cis-trans plot summarizing the NAD-QTL^epi^ mapping results. Only significant linkage associations are shown. X-axes: position of the parental differentially methylated regions; y-axes: position of NAD regions. A *trans*-effect was defined when the NAD target was located outside of the QTL^epi^ confidence interval (2 LOD drop-off method), and *cis* otherwise. Green dots correspond to a positive QTL^epi^ effect, whereas red dots correspond to a negative QTL^epi^ effect. The barplots show the percent of NAD-QTL^epi^ that are either positive or negative and belong to one of the indicated categories. Category “arm-arm”: both the QTL^epi^ and the NAD target region are located on the chromosome arm; category “arm-peri”: the QTL^epi^ is located on the chromosome arm, but the NAD target in the pericentromeric region; category “peri-arm”: the QTL^epi^ is located in the pericentromeric region, but the NAD target on the chromosome arm; category “peri-peri”: Both the QTL^epi^ and the NAD target are in the pericentromeric region. **(B)** The boxplots show the percent of midparent divergence in DNA methylation in the epiHybrids at the NAD targets, categorized by QTL^epi^ effect direction (positive or negative) and predominant NAD remodeling scenario (trans-chromosomal de-methylated or trans-chromosomal methylation). Blue: mid-parent divergence in epiHybrids whose paternal epiRILs were epihomozygous WT (MM) at the QTL^epi^; red: midparent divergence in epiHybrids whose paternal epiRILs were epihomozygous *ddm1* (UU) at the QTL^epi^.

**Figure S9.**
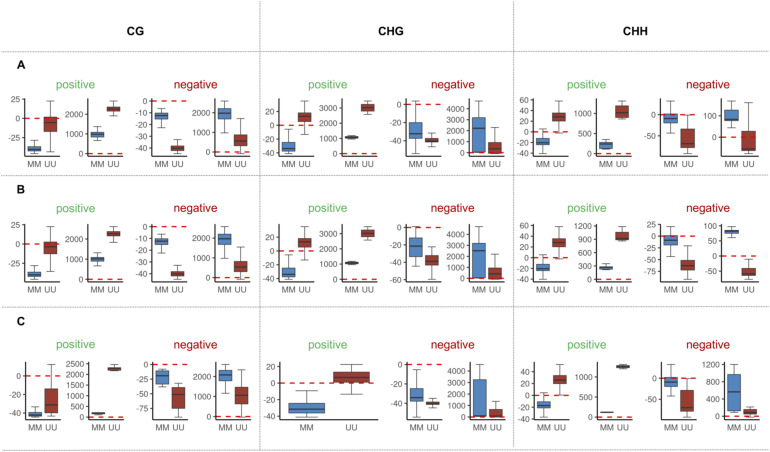
Extension of the boxplots showing the percent of midparent divergence in DNA methylation in the epiHybrids at the NAD targets, categorized by QTL^epi^ effect direction (positive or negative) and predominant NAD remodeling scenario (trans-chromosomal de-methylated or trans-chromosomal methylation). Blue: midparent divergence in epiHybrids whose paternal epiRILs were epihomozygous WT (MM) at the QTL^epi^; red: midparent divergence in epiHybrids whose paternal epiRILs were epihomozygous *ddm1* (UU) at the QTL^epi^. **(A, B, C)** presented for all NAD-QTLs (both in trans and cis), (B) presented for cis NAD-QTLs only, (C) presented for trans NAD-QTLs only.

We found that the majority (94%) of all detected NAD-QTL^epi^ associations occurred at distal regions (see the Materials and Methods section), and were largely confined to pericentromeric regions (88% of total), both intra-chromosomally (90%) and inter-chromosomally (10%), (Fig 6A). To understand the effect direction of these associations in more detail, we partitioned the NAD targets according to whether they are mainly characterised by TCM or TCdM events across epiHybrids (see the Materials and Methods section). For negative associations, which were mostly at distal regions (96%), we found that TCM events in pericentromeric NAD regions were more likely to occur in epiHybrids whose paternal epiRIL parents were methylated at the QTL^epi^ (Figs 6B and S9). Likewise, TCdM events were more likely in epiHybrids whose paternal parents were unmethylated at the QTL^epi^. By contrast, for positive associations, TCM events in NAD regions were more frequent in epiHybrids whose paternal parents were unmethylated at the QTL^epi^, and TCdM events more prevalent in epiHybrids whose paternal parents were methylated (Figs 6B and S9). The NAD regions that were targeted by QTL^epi^ were also strongly correlated with each other across the genome. This result indicates that the QTL^epi^ act pleiotropically and contribute to the genome-wide co-remodeling of distal regions in the epiHybrids.

### Parental pericentromeric DMRs affect transcriptional and phenotypic heterosis

**Figure 7.**
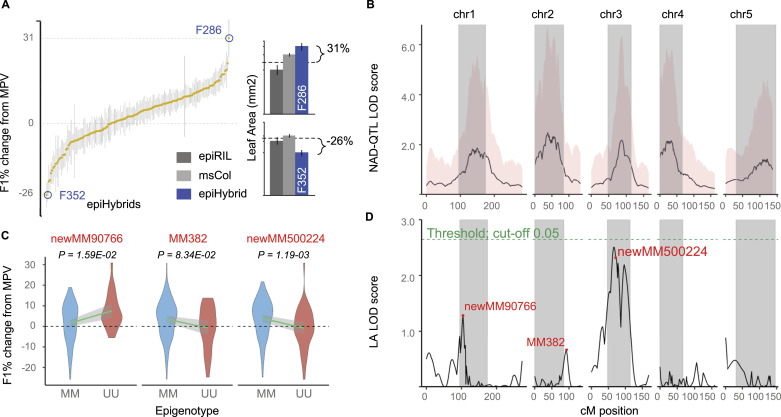
Parental pericentromeric differentially methylated regions are associated with leaf area heterosis. **(A)** Distribution of leaf area (LA) in the epiHybrids expressed as percent change from mid-parent value shown for all 6,912 phenotyped plants (190 epiHybrid families × 6 siblings on average). Yellow dots: mean values per epiHybrid family; vertical lines mark off the minimum and maximum value for siblings within each family. Right panel: two extreme examples of midparent heterosis in LA are shown. **(B)** Average LOD score profile for NAD regions that are uniquely associated with a given QTL^epi^ position (x-axis). Significant QTL^epi^ were mostly detected within core pericentromeric regions of each chromosome. Pink background represents the SD of the NAD-QTL^epi^ LOD profiles along the genome. **(C)** Effect sizes and direction of the detected phenotypic QTL^epi^ (differentially methylated regions: newMM90766, MM382, and newMM500224). Violin plots present LA in the epiHybrids expressed as percent change from mid-parent value as a function of the epigenotypes of the paternal epiRILs at the QTL^epi^ position; MM (epihomozygous wt) or UU (epihomozygous *ddm1*). *P*-values indicated in the figure are from a linear regression (two-tailed *t* test). **(D)** An unbiased phenotypic QTL^epi^ scan for LA heterosis.

Our NAD-QTL^epi^ analysis revealed that parental pericentromeric DMRs have substantial pleiotropic effects on DNA methylation remodeling throughout the genome (Figs 6A and 7B). It is likely that these DMRs also induce phenotypic heterosis in the epiHybrids by perturbing transcriptional regulation at specific genes. To begin to test this, we phenotyped 190 epiHybrid families with 18 siblings on average (6,912 plants in total) using an automated high-throughput phenotyping facility (Klukas et al, 2014; Junker et al, 2015) (see the Materials and Methods section). We chose projected leaf area on the 18th d after sowing (DAS) as our focal trait, as LA has often been used as an indicator of hybrid performance (Meyer et al, 2004). Mid-parent heterosis (MPH) was defined as the phenotypic divergence (in %) of an epiHybrid from the average phenotype of its two parents middle parental value (MPV) (see the Materials and Methods section) (Fig 7A) (Table S7). There was substantial MPH between families, with some epiHybrids displaying up to 31% increase and 26% decrease in LA, respectively (Fig 7A). In total, about 60% of all epiHybrids families displayed significant transgressive phenotypes relative to their parents, either the the form of low parent or high parent heterosis (Table S8). Moreover, variance component analysis indicated that 30% of the total variation in MPH could be explained by between-family variation (see the Materials and Methods section). This latter estimate implies that the contribution of the paternal methylome to each family is a major determinant of heterosis in the epiHybrids. Similar observations were made previously in a much smaller panel of 19 *ddm1-2*-epiHybrids, derived from a different set of paternal parents (Lauss et al, 2018).

Table S7 Results from the phenotyping experiment showing the mid-parent heterosis.

Table S8 Selection of the model explaining heterosis. The model can be either additive or transgressive (best-parent and least-parent heterosis).

**Figure S10.**
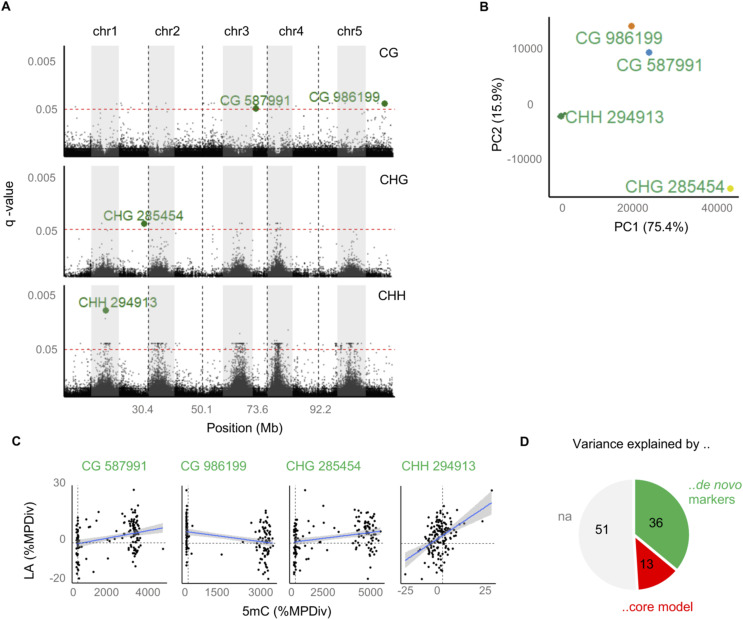
EWAS analysis of leaf area heterosis. **(A)** Manhattan plot showing associations between NAD regions and LA heterosis. Significant associations were identified from a Likelihood Ratio Test between a‚ core model’ and a‚ de novo model’. The core model uses the three parental QTL^epi^ previously identified from the linkage scan (see Fig 5), whereas the de novo model uses the core model plus the methylation mid-parent divergence of a given NAD region as an additional predictor. The *P*-values were adjusted using Benajmini–Yekutieli multiple comparisons and are plotted on the y-scale (*Q*-value). **(B)** Labeled in green are four NAD regions that were selected as most significant (see (B)). **(B)** Hierarchical clustering on principal component analysis of all significant epigenome-wide association study were performed to group and select specific NAD regions (*Q*-value < 0.05) as predictors. The epigenome-wide association study strongly clustered into four groups. For each group we use the most significant NAD region as a proxy predictor in the final “de novo model.” **(C)** Scatter plots show the correlations between mid-parent divergence in LA and midparent divergence in DNA methylation at the four selected NAD regions. **(D)** Variance component analysis was used to estimate how much of the phenotypic divergence of LA can be attributed to parental QTL^epi^ (core model) and to de novo NADs (de novo model).

To assess if the between-family variation in MPH can be explained by parental pericentromeric DMRs, we performed an unbiased genome-wide QTL^epi^ scan using parental DMRs as predictors and the degree of LA MPH within each epiHybrid family as the outcome variable (see the Materials and Methods section) (Fig 7D). Our search identified a borderline genome-wide significant QTL^epi^ on chr 3 and two weak QTL on chr 1 and 2, all of which mapped to pericentromeric regions of these chromosomes. Interestingly, the QTL^epi^ on chr 3 also mapped close to the QTL^epi^ identified by Lauss et al (2018), which was associated with leaf area and flowering time in their smaller pilot study (Lauss et al, 2018). Together these three QTL^epi^ explained ∼12% of the total between-family MPH variance (see the Materials and Methods section) (Fig S10D). Interestingly, on chr 2 and chr 3, the epiHybrids whose paternal epiRIL parent was wt-like at the peak QTL^epi^ marker showed positive heterosis, whereas epiHybrids whose paternal epiRIL parent was *ddm1-2*-like did not (Fig 7C). This observation points at possible epistatic interactions between the wt epihaplotypes at these loci and the epigenomic backgrounds of the hybrids. Indeed, a quantitative (epi)genetic interpretation of the QTL^epi^ effects in the epiHybrid system, reveals that both epistasis and dominance jointly contribute to the detected effects, and cannot be effectively delineated from each other (see the Materials and Methods section). The same holds true for similar experimental designs that have used classical F1 hybrid crosses (Yang et al, 2017; van Hulten et al, 2018).

As can be seen in Fig 7B and D, our LA QTL^epi^ mapped in close linkage disequilibrium (LD) with the pleiotropic NAD-QTLs identified above (Fig 7B and D) (Table S9). Perhaps as a consequence of this co-location, the LA QTL^epi^, themselves, were also highly pleitropic, being associated with 806 NADs in total (locally and at distal regions combined). We reasoned that their association with these NADs could impact the expression levels of proximal genes, and thus provide a molecular mechanism for their impact on leaf area heterosis. A total of 594 of these 806 NADs were indeed located within 1 kb of 355 unique protein-coding genes. To test their effect on expression, we sequenced the transcriptomes of the same 36 trios (72 samples in total) that we had used for sRNA-seq analysis (see the Materials and Methods section). Of the 594, 84% (499) were still significant in the subset of 36 trios, which corresponded to 302 unique genes. Further filtering revealed that 26 of the corresponding genes also had clear correlations in their degree of midparent divergence in expression and DNA methylation, whereas the expression divergence of another 25 genes were directly associated with the LA QTL^epi^ (Table S10). Together, the final list contained 43 unique candidate genes.

Table S9 Results of the linkage disequilibrium analysis between markers of LA QTL^epi^ and pleiotropic NAD-QTLs, using their epigenotype profiles.

Table S10 List of heterosis genes showing a clear relationship between their midparent expression divergence and changes in either DNA methylation or expression related to LA QTL^epi^.

**Figure S11.**
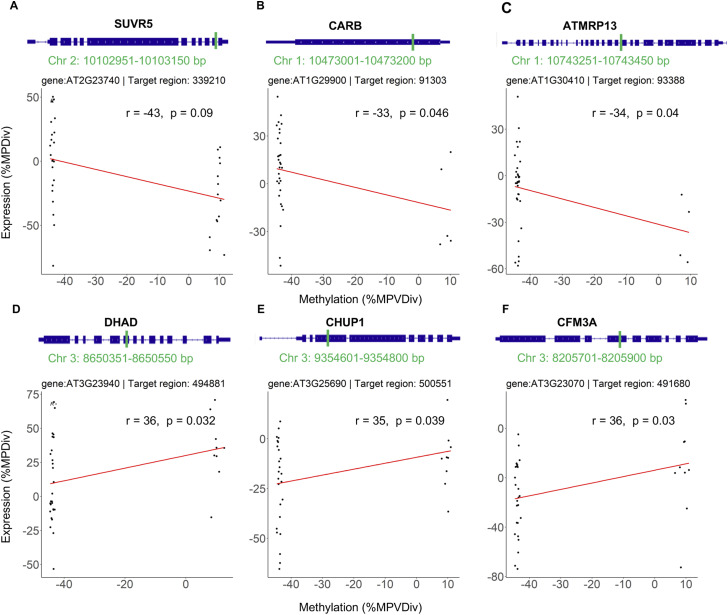
Example of genes showing how epiHybrid methylation divergence (x-axis) correlates with expression change (y-axis). On top of each figure is a browser picture of each gene and in green is indicated the location of the corresponding target region. (A, B, C, D, E, F) display examples of specific genes.

The final list of candidate genes was enriched for epigenetic and stress-response pathways (Table S10). Among the candidate genes associated with the QTL^epi^ on chr3, we identified a chromatin remodeling protein of the CLASSY family (CLSY4), DIHYDROXYACID DEHYDRATASE (DHAD), and CHLOROPLAST UNUSUAL POSITIONING 1 (CHUP1). CLSY4 contributes to locus-specific and global regulation of DNA methylation via controlling the production of 24 nt sRNAs (Zhou et al, 2018). DHAD is involved in salt stress response, with loss-of-function mutants showing increased sensitivity to abiotic stressors and reduced root (Zhang et al, 2015). CHUP1 is crucial for chloroplast movement in leaves in response to light (Oikawa et al, 2003) and impaired of chloroplast movement strongly affects vegetable and reproduction growth (Howard et al, 2020). Among the candidate genes associated with the QTL^epi^ on chr 2, we identified the histone methyltransferase SU(VAR)3-9 RELATED 5 (SUVR5) (Fig S11). SUVR5 is part of a multimeric complex that represses gene expression by altering histone modifications and loss of function mutants exhibiting delayed flowering and reduced root growth (Caro et al, 2012). Cortijo et al also identified a QTL^epi^ associated with root length in a distal location on chr 2 (Cortijo et al, 2014).

**Figure S12.**
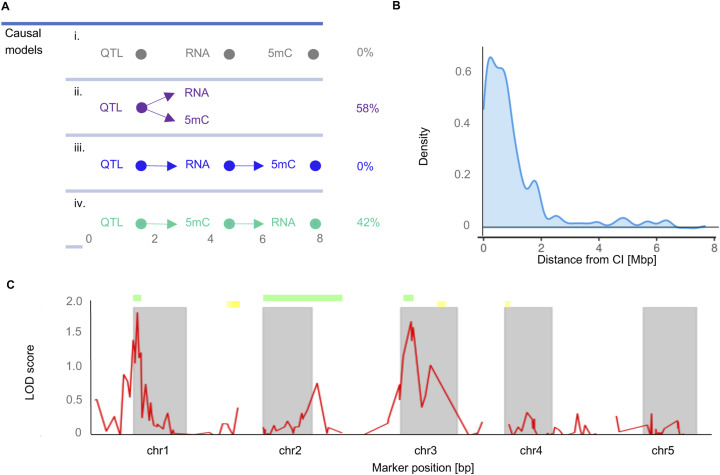
Characterization of detectd QTLepi. **(A)** Description of causal models. Model “i” indicates that there is no direction in the relationship between gene expression, methylation and QTL^epi^. Model “ii” shows that QTL^epi^ act on methylation and gene expression independently. Model “iii” shows how QTL^epi^ act on methylation through gene expression and model “iv” shows how QTL^epi^ acts on gene expression through methylation. **(B)** Distribution of the distances from trans-NAD-QTL targets and their corresponding confidence interval boundaries. **(C)** Genome-wide QTL scan for LA comparing confidence intervals of significant peaks (marked with green) with published QTLs^epi^ for mid-parent heterosis in leaf area from Meyer et al (2010) (marked with yellow).

To obtain first mechanistic insights into how the pleiotropic LA heterosis QTL^epi^ affect these candidate genes, we performed causal modelling (see the Materials and Methods section). We found that for most genes (58%), the pleiotropic QTL^epi^ affect DNA methylation and expression independently. Nonetheless, consistent with Meng et al (2016), a substantial proportion (42%) of QTL^epi^ also affect gene expression indirectly via effects on DNA methylation at proximal NADs (Fig S12A), rather than the latter occurring in an expression-dependent manner (Secco et al, 2015). However, 90% of these latter NAD-QTL^epi^ associations were in at distal regions, with most of the NAD targets being located within 2 Mb outside of the QTL^epi^ confidence interval (Fig S12B). These distal associations therefore require some type of long-range signal by which the QTL^epi^ can affect the NAD status. One possibility is that the differential production of distal-acting sRNA from DMRs within the QTL^epi^ confidence interval leads to differential targeting of the NAD regions. Preliminary support for this comes from the fact that variation in sRNAs among epiHybrids for 13% of the same NAD-QTL^epi^ associations correlate with the QTL^epi^-induced methylation variation of the NAD target regions (see the Materials and Methods section). Follow-up molecular work is required to further delineate a sRNA-based mechanistic model underlying these at distal effects.

## Discussion

Here, we have shown that DNA methylation differences between isogenic parents are sufficient to trigger methylome remodeling and phenotypic heterosis in F1 hybrids. Our multi-family experimental design allowed us to delineate, for the first time, that most of these remodeling events are induced by distally acting pericentromeric parental DMRs. These distally induced methylation changes affect the transcriptional output of a large number of target genes, which collectively contribute to phenotypic heterosis. Although the precise regulatory mechanisms underlying these effects cannot be fully resolved here, our data support a central role for distally-acting sRNA. Regardless of the molecular underpinnings, our work establishes parental pericentromeric DMRs as important predictors of heterosis. Recently, Seifert et al (2018) showed that sRNA differences between maize parental genotypes were strong predictors of heterosis in the hybrid offspring, which is consistent with the pericentromeric origin of these effects. In the epiHybrid system, sRNA differences between the parental lines could originate from the hypomethylation of specific loci in epiRIL paternal parents. This hypomethylation may be accompanied by a loss of 24 nt matching sRNA (Fig 3C) and may lead to non-additive methylation levels at their target regions. To further delineate the role of the RdDM pathway in sRNA-mediated DNA methylome remodeling in the epiHybrids, follow-up heterosis experiments could focus on crosses of specific RdDM mutants (e.g., pol IV) in a *ddm1 or ddm1-epiRIL* background. Manipulating RdDM alone seems to be insufficient to generate heterosis in *A. thaliana* (Zhang et al, 2016).

The methylome remodeling events seen in the epiHybrids may also reflect some type of homeostatic epigenome adjustments in response to specific combinations of hypo- and hyper-methylated regions being forced to co-occur in the same genome. These adjustments are reflected in the stark up- and down-regulation of DNA (de)methylation and heterochromatin-related genetic pathways (Table S10). Whether RdDM is a driver or a consequence of these homeostatic adjustments would have to be determined. However, with knowledge of the relevant epigenetic pathways, their interactions and precise genomic targets, it may be possible to treat hybrid methylomes as the output of a dynamical system, whose steady state and response to perturbations may be amenable to deeper mathematical analysis.

**Figure S13.**
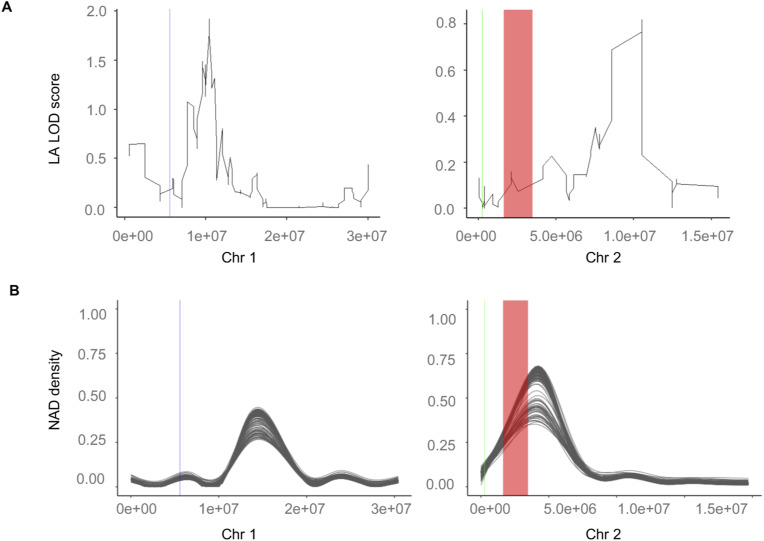
Genetic variation does not affect methylome remodeling in the epiHybrids. With red color is the detected Chr2-2M inversion, with green is the 56-kb inversion, and with blue, the 55-kb inversion, as described in Zhang et al (2023). **(A, B)** The regions are marked in the (A) LA LOD score profile figure from Fig 5A and in the (B) remodeling figure as described in Fig 3A.

Our survey of 169 epiHybrid methylomes also revealed regions harboring de novo NADs, whose origin cannot be easily attributed to locally nor distal-acting parental QTL^epi^ (Fig 6A). Nonetheless, these de novo NADs are shared among families and may therefore have a common, albeit undetected, origin in the paternal methylomes. To explore if these de novo NADs are phenotypically relevant, we performed a conditional epigenome-wide association study for LA MPH in the epiHybrids (see the Materials and Methods section), which controlled for the effects of the already identified parental QTL^epi^ on chr 1, chr 2 and chr 3. This scan identified a large number of significant associations (Fig S10), accounting for about 36% of the between-family variation in LA MPH (Fig S10D). Hence, parental QTL^epi^ in combination with de novo NADs in the epiHybrids explain a major fraction (51%) of the between-family variation in LA MPH, and thus appear to be an important molecular component underlying heterosis. The remaining sources of variation in the epiHybrid system remain obscure. There is currently no evidence that structural variants that have recently been detected in the epiRILs and in close relatives of their *ddm1-2* founder line make any contributions (Fig S13).

**Figure S14.**
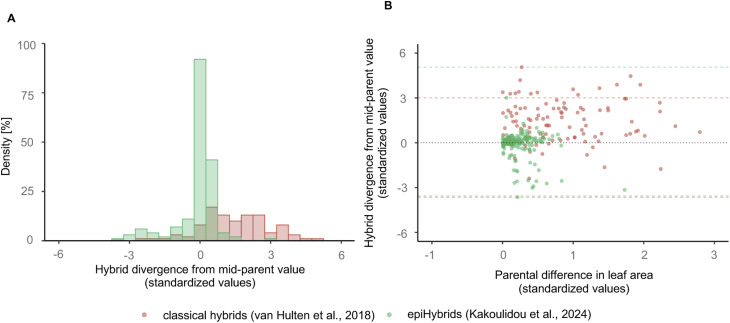
Heterotic effects in the epiHybrids compared with those of a classical hybrid cross design. **(A)** Density plot showing differences of density of hybrid divergence from mid-parental value between classical hybrid study (van Hulten et al, 2018) and epiHybrid study. **(B)** Scatterplot showing the relation between the absolute range of parental divergence (x axis) and the absolute divergence from the mid-parental value (y-axis) for a given epiHybrid and classical hybrid (van Hulten et al, 2018) crosses.

The extent to which parental DMRs contribute to heterosis in classical hybrid crosses where the two parents are also genetically very different is unclear. However, we speculate that many heterosis QTL that have been traditionally attributed to parental genetic polymorphisms may in fact be because of LD with segregating hypo-methylated epialleles that trigger similar methylome remodeling dynamics as observed here. Indeed, many of the heterosis QTL detected in classical Arabidopsis hybrid studies appear to map within pericentromeric regions of chromosomes (80% of all) proximal to our detected QTL^epi^ on chr 1, chr 2, and chr 3 (Fig S12C) (see the Materials and Methods section), which provides some support for this idea. To draw further comparisons between the LA heterosis detected in our epiHybrid system and that of classical F1 crosses using genetically divergent parental lines, we reanalyzed the leaf area data of van Hulten et al (2018) (Fig S14). The authors used a similar crossing design. Although the absolute phenotypic divergence for LA between the parental lines was, on average, much larger in the classical crosses than in the epiHybrid system, we found that the epiHybrids displayed a similar absolute range of transgressive phenotypes compared with the classical F1 hybrids. If similar effects can be generated in crops, epigenomic perturbation strategies may hold breeding potential.

## Materials and Methods

### Plant material

#### The ddm1-2–derived epiRIL population

The *ddm1-2* epiRIL population (Johannes et al, 2009) was obtained from the Versailles Arabidopsis Stock center of INRA. The epiRIL lines were originally generated from two closely related parents of the same accession (Columbia, Col). The male parent was homozygous for the WT in the *DECREASE IN DNA METHYLATION 1* (*DDM1*) allele (Col-wt). The female parent was homozygous for the *ddm1-2* mutant allele (*Col-ddm1*). The resulting F1 of the cross was backcrossed as a female parent to the Col-wt and the progeny plants containing the WT DDM1 allele were selected. After six rounds of single seed descent, a 505 different epiRILs population was propagated. The epiRILs have highly similar genomes, but distinct epigenomes as their DNA methylation variants induced by *ddm1* are stably inherited (Johannes et al, 2009).

#### The msCol plant

To reduce the risk of mistakes during hand pollination or unwanted self-fertilization, a male sterile Col-0 WT line, named msCol, was used as the maternal plant. The msCol line was established by crossing Col-0 with the male-sterile line N75 (Melchinger et al, 2007). Line N75 has a recessive mutation for the male sterility 1 (MS1) gene that controls the development of the anther and pollen in Arabidopsis. Homozygous ms1 mutants cannot produce viable pollen. It is a recessive mutation that is maintained in the heterozygous state (appearing as WT) in a progeny that contains both homozygous mutant seeds and heterozygous WT individuals. The origin of the MS1 gene is in the Ler background. However, the mutant allele of the gene was introduced into a Columbia background by six generations of repeated backcrossing to Col-0. The recurrent Col-0 parent genome was recovered by using marker-assisted selection (Melchinger et al, 2007). The msCol seeds were obtained from the University of Hohenheim. We performed three more rounds of backcrossing at the Leibniz Institute of Plant Genetics and Crop Plant Research (IPK). Seeds from 2 msCol plants of the same generation, named msCol-12 and msCol-16, were used as the maternal plants for the crosses.

### Plant cultivation and crosses

To exclude that differences in the maternal cytoplasm affect the phenotypes in the F1 and to make sure that Ler loci still left in the msCol plants are constant across the different epiRIL crosses, the msCol plants were used as the maternal parent and the *ddm1-2* epiRILs as the paternal parent.

All epiRILs and msCol individuals were cultivated in single-seed pots randomized under long day (16 h light, 8 h dark) conditions (20°C and 60% humidity) in a greenhouse at the IPK. The msCol seeds were the progeny of a cross between a plant with the MS1 mutation in the heterozygous state and a plant in the recessive homozygous state (ms1ms1 × MS1ms1). Only 50% of the progeny should have inherited the male sterile mutation, an outcome which we verified at the inflorescence stage, because the male sterile formed no pollen. We used for the crosses only msCol plants which carried the homozygous recessive mutation for the *ms1* allele. When the first inflorescence matured, the flowers were examined under the microscope. The plants with anthers containing viable pollen were immediately removed to exclude the possibility of cross fertilization. When the inflorescence of the main stem had flowers opened and the stigmata of the msCol plants were receptive, the epiRILs were used as paternal parents to fertilize the msCol plants. The pollinated female inflorescence formed the epiHybrid progeny, whereas the paternal plants used for the pollination were left to dry with six siliques per plant (Meyer et al, 2004) and were used as the male epiRIL parents in the phenotyping phase. Representative heterozygous siblings of both msCol-12 and msCol-16 were used to pollinate corresponding homozygous male sterile plants and their progeny was used in the phenotyping screening later as the maternal parent.

### Phenotyping screen

Phenotyping and imaging were performed in the IPK HT phenotyping facility for small plants using mobile carriers (Junker et al, 2015). We selected 190 random epiRIL families that do not substantially overlap with epiRILs that were used in previous publications. These epiRILs with their corresponding 190 epiHybrids and the recurrent maternal msCol-0 lines were grown and phenotyped in three replication experiments. In each of the three cultivation experiments, six individuals per line were grown (2,304 individuals per cultivation experiment; 6,912 individuals in total across the three cultivation experiments) on six-well trays filled with a mixture of 85% (v) red substrate 2 (Klasmann-Deilmann GmbH) and 15% (v) sand and covered with black rubber mat, until 27 d after sowing. Plants were imaged daily for vegetative growth and developmental traits.

### Imaging and image analysis

During the cultivation period in the automated system, top and side view images were taken by the RGB (visible light) and fluorescence cameras (Junker et al, 2015). Image-based plant feature extraction was performed using the Integrated Analysis Platform open-source software for high-throughput plant image analyses (Klukas et al, 2014). Leaf area measured as the projected leaf area in pixels under fluorescence light on DAS 18 was used to study heterosis.

### DNA and RNA sample preparation

All epiHybrids and their parental lines were harvested at 27 DAS in a time frame of 3 h as described in Zhang et al (2021). Rosettes of all three replications were pooled together, were immediately frozen in liquid nitrogen, and stored at −80°C until processing. The DNeasy plant mini kit from QIAGEN was used to extract genomic DNA from 169 families for WGBS. DNA and RNA material of 169 epiHybrids, their corresponding 169 epiRIL parents, and the 2 msCol maternal lines were sent to the Beijing Genome Institute (BGI) for WGBS library preparation. Sequencing was performed on an Illumina HiSeq X 10 instrument. Clean raw paired-end files were obtained from BGI and used for downstream analysis. Total RNA was extracted from 36 epiHybrids and their corresponding parents using the miRNeasy kit (QIAGEN) and was sent to BGI for RNA-seq and DNBSEQ UMI Small RNA library preparations. Sequencing was performed on the DNBSEQ platform. The selection of the 36 families was based on their phenotyping performance for leaf area. We chose 12 epiHybrids with the highest divergence from the average parental performance, 12 with the lowest, and 12 that had no difference compared with their parents.

### WGS analysis to detect SNPs in the msCol plants

Genomic DNA from the 2 msCol lines was provided to the BGI for whole genome re-sequencing. Clean raw reads obtained from BGI were quality-trimmed using Trimmomatic (Bolger et al, 2014) in the paired-end mode (LEADING:5 TRAILING:5 MINLEN:50 SLIDINGWINDOW:3:18).

Sequences were mapped to the reference genome using Bowtie2 (Langmead & Salzberg, 2012). Duplicate reads were marked and sorted using Picard. Variant calling was performed with GATK (Van der Auwera and O’Connor, 2020) to identify polymorphisms between the available Columbia genome and the variants were provided as input for a base quality score recalibration. SNPs and INDELs were extracted using GATK and processed separately. We removed low-quality sites (QUAL > 40) and filtered for homozygotes.

The second round of variant calling was performed using the recalibrated bam file. SNPs and INDELs were annotated using snpEff (Cingolani et al, 2012). We treated the two msCol siblings (msCol12 and msCol16) separately and we found the reported overlap by using Bedtools (Quinlan & Hall, 2010) intersect.

### Methylome mapping and characterizing hybrid remodeling

The WGBS data of the epiHybrids and their parental lines were processed using Methylstar (Shahryary et al, 2020). Summary statistics can be found in Table S1. Using jDMR (Hazarika et al, 2022) we divided the genome into sliding 200 bp regions with a step size of 50 bp and bins with at least 10 cytosines (Tables S2, S3, and S4). In the parental lines, any given 200-bp region was classified either as unmethylated or methylated, whereas the F1 epiHybrids were classified as methylated, unmethylated or intermediate methylated. A final binary matrix with each region state call was created. “0” indicates an unmethylated region, “1” a methylated region, and “0.5” an intermediate methylation state. Using this binary matrix, for each epiHybrid and its corresponding parents, we calculated the parental divergence (PD), the MPV, and the epiHybrid divergence from the MPV (HD) at each 200 bp region. PD was calculated by subtracting the methylation state call of the epiRIL from the methylation state call of the msCol. The MPV was calculated as the average of both parents ((msCol + epiRIL)/2). The HD was calculated by subtracting the MPV from the methylation state call of the F1 hybrid. Non-additivity (NAD) in one region is inferred when epiHybrid divergence differs from zero and additivity when it equals zero. A DMR is presumed when the PD at a given region differs from 0 and SMR when the PD is equal to 0 (Tables S5 and S6).

### Enrichment of annotations

jDMR (Hazarika et al, 2022) was used to annotate the regions of interest and the updated annotation files for genes were downloaded from Ensembl Plants.

### Enrichment of DMRs among remodeling scenarios

To demonstrate the substantial enrichment of DMRs within various remodeling scenarios, we performed a bootstrapping analysis across all epiHybrid families. We conducted 1,000 random samplings by selecting regions identified by jDMR and calculated the frequency of remodeled DMRs within all non-additive regions. The distribution of these frequencies is presented in Fig S3C. Subsequently, the *P*-value was determined from this empirical distribution, using the cut-off for global frequency of remodeled DMRs.

### DMR call to detect DMRs between the msCol plants and the Col-0 reference

To verify that the msCol methylome strongly resembles a Col WT plant methylome, we identified DMRs between the msCol and a publicly available Col plant, the Col0 G0 MA3 line ([Shahryary et al, 2020]; GEO accession number GSE153055). As a control group, we dissected DMRs between the same Col0 GO MA3 line and another published Col line ([Yang et al, 2016]; Col-0 replicate 1; GSE70912). To identify these regions, we used jDMR as described in Hazarika et al (2022).

### RNA-seq and sRNA analysis

RNA-seq data were analysed on the clean FASTQ data obtained from BGI. Low-quality sequences were removed using Trim Galore and aligned to the reference *A. thaliana* (TAIR10) genome using Tophat2 (Kim et al, 2013). Reads were counted using featureCounts (Liao et al, 2014) and the resulting raw count table was used downstream in R. Raw counts of genes with at least one counts per million in at least two samples were kept and normalised using the TMM method of edgeR package (Robinson et al, 2010). Using the normalised counts, we computed the epiHybrid’s expression divergence as (epiHybrid − MPV)/MPV × 100.

Analysis of the obtained clean sRNA data was done using Shortstack with default parameters (Johnson et al, 2016): ShortStack -readfile X_epiRIL.fq.gz -outdir X_epiRIL -genomefile TAIR10_chr_all.fa -nohp -locifile 200 bp_regions_per_context.csv. “-nohp” disables MIRNA search, whereas “-locifile” specifies intervals to be analysed. As intervals, we used our 200-bp regions and ran for each line Shortstack once for each context. The output Result file for each line has the raw reads counts at each locus for each sRNA size length separately (short, 21, 22, 23, 24, long). Read counts were normalised using the TMM method of the edgeR (Robinson et al, 2010) package and used downstream.

### Correlations

To quantify a linear relationship between mid-parental methylation divergence of 200-bp regions, we performed a correlation analysis for regions that shared a remodeling scenario (TCdM or TCM) for at least 10 epiHybrid families. We filtered out significant associations (*P* < 0.05), together with their Pearson’s Correlation Coefficient. We selected a set of pairwise correlations, in which both regions were represented by one of the remodeling scenarios for at least 50% of the epiHybrid families.

### QTL mapping analysis

Based on the set of the 200-bp regions, we selected NADs that shared a remodeling event (TCdM or TCM) for at least 50% of the epiHybrid families. We normalised the degree of mid-parental methylation divergence of NADs with the Ordered Quantile transformation (Peterson & Cavanaugh, 2019). We used a recently updated recombination map of the epiRILs consisting of 144 stably inherited DMRs (Zhang et al, 2021). At any given DMR an epiRIL is either epi-homozygous for the WT methylated state (MM) or epi-homozygous for the ddm1-like-state (UU). We used these DMRs as physical markers together with the normalized degree of mid-parental methylation divergence as phenotype trait in the classical interval mapping approach implemented in scanone function from R/qtl package (Broman et al, 2003). The mapping was performed with a step size of 2 cM and estimates were obtained by Haley-Knott regression. Genome-wide significance was determined empirically for each trait using 1,000 permutations of the data. LOD significance threshold corresponds to a genome-wide false positive rate of 5%. For each NAD, we selected the highest peaks per chromosome, provided it passed the genome-wide LOD significance threshold. To adjust for multiple testing across NADs we used the Benjamini-Yekutieli correction (FDR < 0.05).

To quantify the NAD-QTL^epi^ effect we used the R^2^ value of a linear regression model with the QTL^epi^ as predictor and the NAD (methylation divergence) as the response variable. The linear regression slope (a > 0; positive or a < 0 negative) defined the effect direction. A positive effect direction meant that when two parents were differentially methylated, the hybrid had an increased mid-parent divergence compared with the homozygous methylated parents. A negative effect direction indicated that when two parents were differentially methylated, it led to a negative mid-parent divergence compared with the homozygous methylated parents.

To distinguish local from distal-acting effects we took the following approach: For each NAD-QTL^epi^ we obtained the confidence intervals (CI) around the peak QTL position using a 2 LOD drop-off criterion. If the body of the NAD target was located within a given CI, the NAD-QTL was defined as locally-acting (*cis*), otherwise as distally-acting (*trans*).

### Quantitative (epi)genetic interpretation of the QTL effects

In the construction of the epiHybrid populations we employed an asymmetrical cross design, insofar that all epiRIL parental lines were crossed to a recurrent Col-wt parent. Moreover, for QTL mapping, we defined the phenotype as the divergence from the mid-parent value and subsequently treated the different F1 crosses as a single mapping population. This raises the question to whether the detected QTL effects are because of dominance action of the underlying loci, or because of effects such as additivity or epistasis.

To explore this issue analytically, suppose there are *Q* independent loci determining mid-parent heterosis value z. Let *N* = *Q* − 1 be the number of loci excluding locus *l*, which we will consider as the focal QTL whose phenotypic effects we wish to evaluate. We assume that a proportion 1-*p* of the *N* background loci are *UU* in a randomly chosen epiRIL parent, and *p* are *MM* (here *M* is a methylated allele and *U* an unmethylated allele). The expected midparent value, *mid*, conditional on the fact that a randomly chosen epiRILs parent is *MM* at locus *l* is

$$E\left(mid\;|\;l=MM\right)=\frac{E\left(y_{wt\;}\right)+E\left(y_{epi}\;|\;l=MM\right)}{2}$$

and conditional on locus *l* being *UU* it is

$$E\left(mid\;|\;l=UU\right)=\frac{E\left(y_{wt\;}\right)+E\left(y_{epi}\;|\;l=UU\right)}{2}$$

The expected mid-parent heterosis value *z* for randomly chosen epiHybrid conditional on the fact that locus *l* was *MM* in the parental epiRIL is3

$$E\left(z\;|\;l=MM\right)=E\left(y_{F1}\;\left|\;l=MM)+E(mid\;\right|\;l=MM\right)$$

and similarly, the expected mid-parent heterosis value *z* for randomly chosen epiHybrid conditional on the fact that locus *l* was *UU* is

$$E\left(z\;|\;l=UU\right)=E\left(y_{F1}\;\left|\;l=UU)+E(mid\;\right|\;l=UU\right)$$

The QTL effect in the epiHybrids is given by the contrast

$$QTL_{F1,l}=E\left(z\;|\;l=MM\right)-E\left(z\;|\;l=UU\right)$$

where the conditionality refers to the epigenotypes of the epiRIL parent lines, rather than the epigenotypes of the F1 hybrids. Considering the definitions given in tables below, and assuming equal effect sizes across all of the *N* background loci, it can be shown that the QTL contrast is

$$QTL_{F1,l}=2\beta_{l:D}N\left(\beta_{l:A\;x\;A\;}\left(p-1\right)-2\left(p\left(\beta_{l:A\;x\;D}+\beta_{l:D\;x\;A}-2\beta_{l:D\;x\;D}-\beta_{l:A\;x\;D}+\beta_{l:D\;x\;D}\right)\right)\right)$$

Because the parameter *p* is difficult to determine experimentally and the effect sizes arising from background epistasis are difficult to distinguish from the number of epistatic interactions, we integrate out *p* and replace *Nβ* with *β*, which yields

$$QTL_{F1,l}^{∙}=\overset{1}{\underset{0}{\int }}QTL_{F1,l}dp=2\beta_{l:D}-\frac{1}{2}\beta_{l:A\;x\;A}^{∙}+2\beta_{l:A\;x\;D}^{∙}-2\beta_{l:D\;x\;A}^{∙}$$

**Figure:**
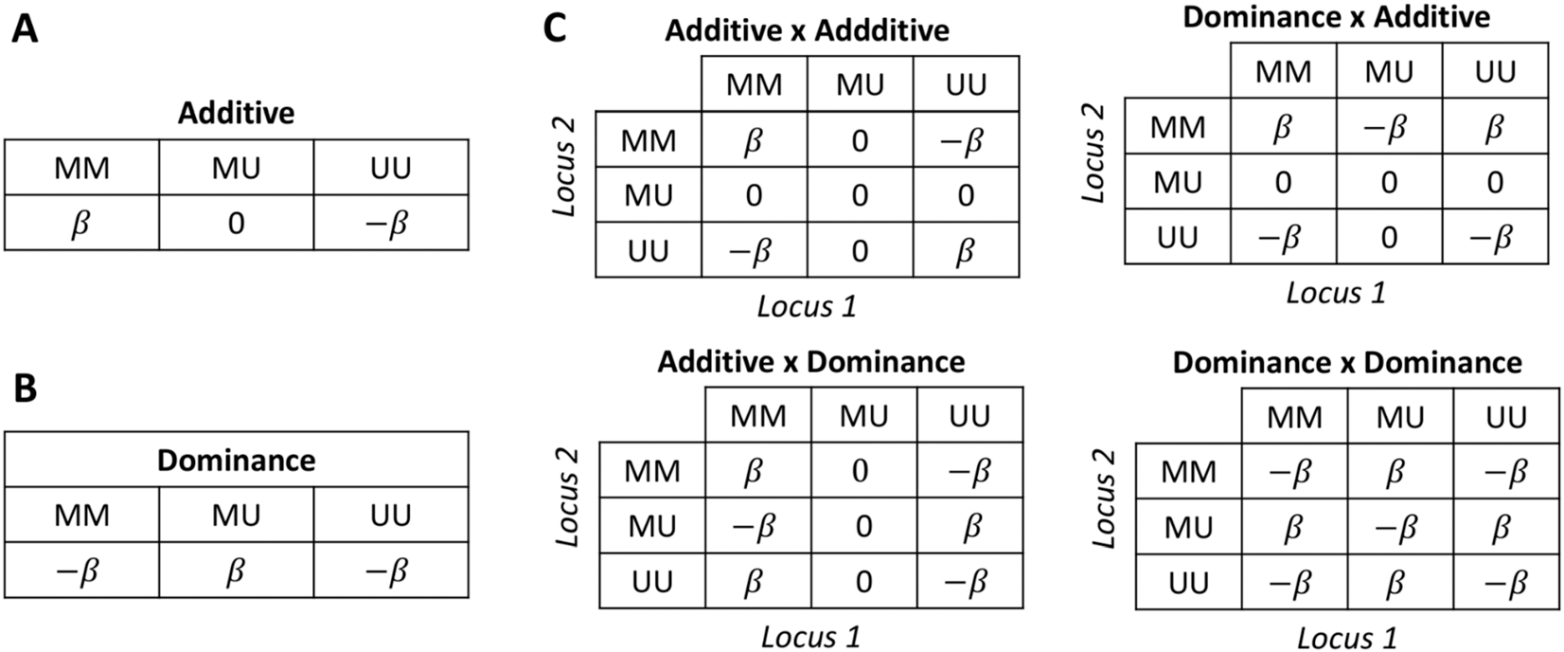


This equation means that the QTL contrast contains a dominance effect via *β*_*l*:*D*_, but also additional effects arising from epistatic interactions between locus *l* and the entire (epi)genomic background (via $\beta_{l:AxA}^{∙}$, $\beta_{l:AxD}^{∙}$ and $\beta_{l:DxA}^{∙}$). Here *A x A*, *A x D* and *D x A* refer to additive × additive, additive × dominance and dominance × additive interactions. Whereas the relative contributions of the dominance and epistatic terms can only be determined experimentally, for example, by help of introgression lines, the effect does require that causal variants are present in the QTL intervals. The causal variants can be in the form of DMRs that are in approximate LD with the peak QTL marker, or else rare structural variants, such as those having arisen from TE mobilization events in the *ddm1-2* founder parent.

### Analysis of heterosis

We normalized raw leaf area measurements on DAS 18 by removing outliers (>3 SD). Families where either the epiRILs or the epiHybrids had fewer than five plant individuals across the experiments were removed. To account for environmental variation, we used a mixed model, using as fixed factor effects germination date, replicates within the experiments, set of experiments, and blocks (eight carriers moving together always in the phenotypic facility). The output model residuals were used to calculate heterosis by using a likelihood approach (Lauss et al, 2018), as the epiHybrid’s percentage change from the MPV: (epiHybrid mean − MPV)/MPV × 100. MPV was defined as the average of both parents (Table S7).

### Variance components analysis and conditional epigenome-wide association study

To search for quantitative trait loci underlying mid-parental heterosis in the leaf area, we performed a classical interval mapping approach with the same recombination map, functions, and hyperparameters from the NAD-QTL^epi^ analysis. We selected the peak QTL^epi^ markers that showed a statistically significant association with LA heterosis (ANOVA; *P*-value < 0.05). To estimate the total heterosis variance explained by the detected QTL^epi^, we created a multiple regression model, named “core model,” which included all detected QTL^epi^ as predictors and percent mid-parent divergence in LA as the response variable. We then specified an alternative model that included the core model plus the mid-parental methylation divergence of a given “de novo” NAD as an additional predictor. These “de novo” NADs were those NADs that did not show an association with parental DMRs in our previous NAD-QTL^epi^ analysis. The goal was to test if such “de novo” NADs explain heterotic variance beyond what is already captured by the core model. In this procedure, each nested model was compared with the core model to show the effect of NADs on heterotic variance. We quantified the improvement in the explanation of phenotypic variance with a Likelihood Ratio Test. We adjusted the *P*-values from likelihood ratio test by Benjamini–Yekutieli correction. We extracted the NADs from the nested models that were statistically better than the core model (FDR < 0.05) and used their mid-parental methylation divergence in hierarchical clustering on principal component analysis. We found that significant NADs form four tight clusters. For each of the four hierarchical clustering on principal component analysis clusters, we selected a single NAD as a proxy in subsequent variance component analysis. We selected that NAD per cluster that explained the most heterotic variance compared with all other NADs in that cluster. Finally, we performed variance component analysis of LA heterosis using a multiple regression model that included both the core model plus the four selected proxy NADs.

### Variation in sRNAs correlates with methylation variation at the NAD-QTL targets

We identified three pleiotropic QTL^epi^ that corresponded to 499 NAD targets. For each of these NAD-QTL^epi^ targets, we calculated the epiHybrids’ methylation and 24 nt small RNA divergence for the 36 trios. Regions that had no 24 nt small RNA reads in any of the corresponding epiHybrids, epiRILs and msCol were removed. For the remaining 199 NAD targets that had reads mapping at these locations, we correlated methylation and sRNA mid-parental divergence. 13% of the correlations were significant with a *P*-value < 0.05.

### Causality

To perform a causal modelling approach, we prepared a set of NAD-QTL^epi^ targets that showed either a significant correlation between the NAD’s mid-parental expression and methylation divergence or the NAD’s mid-parental expression divergence and the QTL^epi^. We filtered out genes that shared at least one of the filtered NAD-QTL^epi^ targets. For each gene, we collected the markers’ epigenotype, targets’ midparental methylation, and expression divergence of the given NAD-QTL^epi^ association. We considered causal relationships where QTL^epi^ acts on methylation through gene expression, QTL^epi^ acts on gene expression through methylation and QTL^epi^ acts on methylation and gene expression independently. We represented these models in the form of acyclic graphs to perform a log-likelihood-based approach implemented in the GraphicalModels package (Schadt et al, 2005). The best-fitting causal model was chosen among 1,000 bootstrap samples according to the Akaike Information Criterion.

### Classical Arabidopsis heterosis studies map proximal to our detected QTLs

For each heterotic QTL^epi^ we obtained the confidence intervals (CI) around the peak QTL position using a 1 LOD drop-off criterion and compared them with the leaf area QTLs published in Meyer et al (2010). In this latter study, they performed an image analysis (Walter et al, 2007) of leaves from Col-0 and C24 parental families of *Arabidopsis* plants harvested on the 6, 8 and 10 DAS.

## Data Availability

Sequence data produced for this study have been deposited in the NCBI GEO database under GSE211719. Sequence data for the *ddm1* mutant (Fig S3) were downloaded from GSM1014117 (Zemach et al, 2013; Zemach et al, 2013). For calling DMRs, G0 MA3 line (Shahryary et al, 2020) was obtained from GEO accession number GSE153055 and Col-0 replicate 1 (Yang et al, 2016) from GSE70912.

## Supplementary Material

Reviewer comments
